# A proposed equation for calculating the total horizontal distance in projectiles of varying launch and landing levels

**DOI:** 10.12688/f1000research.140934.2

**Published:** 2024-03-13

**Authors:** hussein omer

**Affiliations:** 1 Physical Education & Sports Sciences, University of Al-Qadisiyah, Al Diwaniyah, Iraq, 58001, Iraq

**Keywords:** track and field, different levels, biomechanics, launch angle

## Abstract

**Background:**

The projectile draws a path for the flight of the tool, a horizontal distance can be calculated according to the Galileo Galilei Law of Projectiles (GGLP), but only if the two points of the launch and fall of the tool are equal, otherwise we need an equation to be added to the (GGLP) to calculate the real distance that was generated due to the difference between the launch and fall points. There are several equations to calculate this, but they are complex and can be simplified.

**Methods:**

The proposed equation was tested by exporting samples from three different throwing events (javelin, shotput, disc) data in track and field games, to calculate the horizontal throwing distance. The proposed equation was based on the basics of mathematics and geometry. The equation was tested in terms if the height is zero, the proposed equation is suitable even for projectiles with equal levels, and the credibility of the proposed equation with the previous equation was tested statistically.

**Results:**

It was found that there were no differences between the two equations (p>0.05). and due to the relative ease of access of the proposed equation to very similar results, researcher suggests applying the proposed equation.

**Conclusions:**

The proposed equation contained the height factor in the previous equation, and when tested by several criteria, the proposed equation has proven its credibility, statistically and graphically. The ranges of theoretical achievement calculated by the proposed equation are often closer to the real achievements.

## Introduction

Throwing activities are a part of track and field games activities, in which the achievement depends on the horizontal distance covered. It is cut off by the tool thrown from the shooter’s hand, and because the device is thrown, it is subject to the Galileo Galilei Law of Projectiles (GGLP). From a practical point of view, the shell draws a path for the flight of the projectile. Theoretically, it is possible to calculate the horizontal distance it will cover through three essential factors (height, angle and velocity of the projectiles). In GGLP there are only two factors that we can observe, namely the angle and velocity of throwing tools ( Hall, 1995).

$$\text{Horizontal distance}=\frac{v^{2}\sin2\theta }{g}$$

Where ( *v*) velocity of release, ( *θ*) angle of release, ( *g*) acceleration due to gravity.

GGLP measures the best angle to achieve the best horizontal distance (45 degrees) when the launch and landing levels are equal. However, in throwing events in track and field games, the initial starting height is always greater than the final height. Here the launch angle will vary, and the role of the third factor appears, which is the height difference between the throwing tool when launched, which requires that some adjustments be made to GGLP to calculate the horizontal distance to be accomplished theoretically. The resulting value is close to the measurement on the ground. The basis of the adjustments depends on adding a difference in the height of the launch from the landing to GGLP, to calculate the remainder of the distance. The case was treated according to the rule above ( Hall, 1995).

$$\text{Horizontal distance}=\frac{v^{2}}{g}\;\text{Cosθ}\;(\text{sinθ}+\sqrt{\sin2\theta +\frac{2\mathrm{gh}}{v^{2}}})$$

Where ( *v*) velocity of release, ( *θ*) angle of release, ( *g*) acceleration due to gravity, ( *h*) height of release

We also see another formula for the additive equation, which is ( Kinomura *et al.*, 2013). The law has been written in several forms, including:

$$\text{Horizontal distance}=x_{0}\frac{v_{0\;\sin\theta_{v}\;\cos\theta_{v}+v_{0}\;\cos\theta_{v}\sqrt{{\left(v_{0}\;\sin\theta_{v}\right)}^{2}+2gy_{0}}}^{2}}{g}$$

Where ( *x*) initial horizontal, ( *v*) velocity of release, ( *θ*) angle of release, acceleration due to gravity, ( *y*) height of release

Among the studies that dealt with analyzing the content of tournaments are Ariel *et al.* (1997); BADURA (15–23 August 2009); Abdel-Monsef Aly *et al*. (2012), and Mercadante *et al.* (2007).

The disc release angle varies in the effectiveness of the disc in normal conditions (36-38 degrees), meaning it is less than the best angle (45 degrees) due to the height of the shooting point. The best angle for the javelin (37-38 degrees) and the ideal angle (score 41-38) ( Omer, 2018).

The aim of this study is to propose an equation to simplify the previous equation and reduce the steps required to reach the result.

The hypothesis of this research proves that the proposed equations have similar results compared with the previous equations.

## Methods

Sixteen Players from the Jung *et al*. (2012) and Woen-Sik Chae (2011) championships of javelin throwing events have been tested, males and females. Twenty eight players of the discus throw event ( Panoutsakopoulos, 2008; Finals, 2011), males and females and thirty two players of the shotput throw event ( Jung *et al*., 2012) males and females. These sources relied on video recordings and movement analysis using special software to extract velocity, angle, and height of projectile.

The studies from which the data were taken were used high-speed digital video camera and programs were used to analysis motions. The three variables to the projectile’s velocity, angle, and height were substituted into the proposed equation. Then the horizontal distance resulting from the two equations was compared statistically, as well as to the actual results that were measured on the, ground in those championships.

The height difference (h) between the launch of the projectile and its fall is the third factor of importance after the velocity and angle of release. The above equations are complicated (used velocity and gravity twice, sine and cosine also) and can be simplified while reaching the same results with high credibility; the simplification method depends on calculating the distance beyond the parabola and assuming that the launch angles are equal to the parabola angle of incidence.

**Figure 1.**
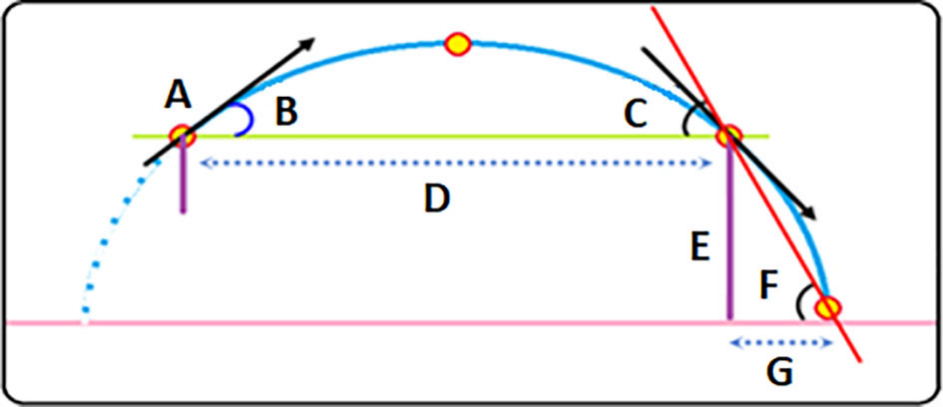
A-Projectile. B-Projectile release angle. C-Projectile falling angle from Parabola path. D-Horizontal parabola distance. E- Release height. F-Falling angle on ground. G-Add distance. D+G final distance.

Figure 1 shows the hypothesis to simplify the equation added to the GGLP to calculate the remaining distance because the throwing tool (release) is higher than the level of its fall.

The proposed simplification method for calculating the remaining distance is based on a simple equation that we add to GGLP according to the following:

We need an angle to calculate the added horizontal distance because the vertical space is known from the launch height according to the law of triangles; given the opposite and adjacent, the other side’s value can be found using the tangent of the pitch. The contrast here is the height of the launch from the ground, and the adjacent is the added distance; the reason for our adoption of this angle is based upon; the falling angle at the moment of changing the path after the tool leaves the parabola path remains the same. Accordingly, the sequence of building the equation is as follows:

$$\tan\emptyset =\frac{\text{Opposite}}{\text{Adjacent}}$$ (1)

$$\mathrm{Add}\;\text{Distance}=\frac{\text{Projection height}}{\tan\emptyset }$$ (2)

$$\text{Horizontal distance}=\frac{v^{2}\sin2\emptyset }{g}+\frac{\text{Projection height}}{\tan\emptyset }$$ (3)

The proposed formula agrees with the following:

1. The advantage of deleting the height. If the height is zero, the proposed procedure is valid even for equal projectiles that have the same shooting and falling heights without problems.
2. To test the reliability of the proposed equation with the previous equation statistically, it has been applied to the data of activities throwing (disc, shotput, and Javelin), whose data has been approved by multiple championships. It is characterized by different heights, tool weights, velocities, and angles, and it was found that the differences were not significant.
3. A virtual test of the graph of the differences between real achievement (measured on the ground) according to the common law and achievement that were also performed theoretically according to the proposed and previous equations. It turns out that the graphic curve of the two equations is harmonious and inverted.
4. A comparison was made between the actual measurement recorded on the ground and the theoretical result from the common law of the proposed and previous equations.
5. The proposed equation is designed to measure the distance from the highest level to the lowest level and has not been tested for anything else.

## Results

The variables were compensated for GGLP and the proposed equation (Performance -Hussein), and the previous equation (Performance-Otto), and the graphs show the horizontal distance resulting from the GGLP and the two equations to observe the phenomenological differences through the raw data.

Table 1 shows the differences in the actual achievement that was measured on the ground and the theoretical achievement that was produced using the general law and the proposed and previous equations in the release variables in the effectiveness of discus throw (n = 28).

**Table 1.** Descriptive statistics variables for discus throw (n = 28).

Variables		Minimum	Maximum	Mean	Std. Deviation
Real achievement (m.)		56.38	69.43	63.442	3.737
Release variables	Height (m.)	1.20	2.05	1.726	0.190
	Velocity (m/s)	23.10	30.80	25.280	2.126
	Angle (deg.)	21.90	39.00	33.496	4.821
Achievement by (m.)	(GGLP)	50.25	74.13	58.858	6.158
	Otto	52.97	76.61	61.397	6.296
	Hussein	53.08	76.69	61.510	6.299
The difference in achievement theoretical and real (m.)	(GGLP)	-11.53	12.34	4.584	6.114
	Otto	-9.61	14.01	-2.045	6.257
	Hussein	-9.48	14.09	-1.934	6.262
The difference between the two equations (m.)	Otto-Hussein	0.06	0.20	0.113	0.039

There are some significant differences between the actual achievements and results from GGLP because the public law measures distance without calculating the difference between launch and fall position, and the two equations are designed to measure the horizontal lengths of projectiles by calculating the difference between launch and fall position. The difference between the two equations is less than (0.12 m.), and the difference between real achievement and Hussein equations is less than Otto equations.

Table 2 shows the differences in the actual achievement that was measured on the ground and the theoretical achievement that was produced using the general law and the proposed and previous equations in the launch variables in the effectiveness of javelin throwing (n = 16).

**Table 2.** Descriptive statistics variables for javelin throwing (n = 16).

Variables		Minimum	Maximum	Mean	Std. Deviation
Real achievement (m.)		59.27	86.27	74.610	9.947
Release variables	Height (m.)	1.78	2.08	1.926	0.091
	Velocity (m/s)	24.42	29.90	26.818	1.489
	Angle (deg.)	31.50	39.40	36.288	2.671
Achievement by (m.)	(GGLP)	59.59	87.86	69.782	7.426
	Otto	61.76	90.42	72.332	7.525
	Hussein	61.84	90.50	72.427	7.523
The difference in achievement theoretical and real (m.)	(GGLP)	-12.62	15.72	4.828	7.998
	Otto	-13.04	15.00	-2.278	7.810
	Hussein	-12.93	15.07	-2.183	7.794
The difference between the two equations (m.)	Otto-Hussein	0.07	0.15	0.094	0.024

Table 3 shows the differences in the actual achievement that was measured on the ground and the theoretical achievement that was produced using the general law and the proposed and previous equations in the release variables in the effectiveness of shotput throwing (n = 32).

**Table 3.** Descriptive statistics variables for shotput throwing (n = 32).

Variables		Minimum	Maximum	Mean	Std. Deviation
Real achievement (m.)		18.74	22.03	20.464	0.924
Release variables	Height (m.)	1.84	2.43	2.086	0.146
	Velocity (m/s)	12.70	14.10	13.369	0.389
	Angle (deg.)	30.80	39.60	35.778	2.371
Achievement by (m.)	(GGLP)	15.34	19.35	17.236	1.079
	Otto	17.77	21.95	19.767	1.135
	Hussein	18.15	22.30	20.142	1.135
The difference in achievement theoretical and real (m.)	(GGLP)	1.95	5.13	3.227	0.923
	Otto	-2.67	0.25	-0.698	0.881
	Hussein	-2.29	0.59	-0.322	0.863
The difference between the two equations (m.)	Otto-Hussein	0.24	0.57	0.376	0.070

Table 4 shows that the differences were not significant. Between the two activities, discus and javelin throwing, when conducting the theoretical calculation to measure the distance using GGLP and the two equations, the probability value was more significant than (p > 0.05), which means that the difference was random.

**Table 4.** ANOVA test between 3 theoretical achievement distance mustering (GGLP, Otto, Hussein).

Events	Source of variance	Sum of Squares	df	Mean Square	F	Sig.
Discus	Between Groups	125.897	2	62.948	1.611	0.206
	Within Groups	3165.230	81	39.077		
	Total	3291.127	83			
Javelin	Between Groups	72.023	2	36.011	0.642	0.531
	Within Groups	2525.554	45	56.123		
	Total	2597.577	47			
Shotput	Between Groups	159.867	2	79.933	64.100	0.000
	Within Groups	115.972	93	1.247		
	Total	275.838	95			

As for throwing the shotput, the differences were significant (p < 0.05) and the reason for that is the weight of the shotput, which causes a decrease in the velocity of its launch because the factor velocity, is squared in the equations.

Table 5 shows least significant difference between the two equations were not significant. and that the distance measurement in the (GGLP) differs from what it is in the two equations.

**Table 5.** LSD test between 3 theoretical achievements in shotput throwing.

Comparing variables		Mean Difference	Std. Error	Sig.
(GGLP)	Otto	-2.53056 ^*^	0.27917	0.000
(GGLP)	Hussein	-2.90569 ^*^	0.27917	0.000
Otto	Hussein	-0.37513	0.27917	0.182

It is noted from the Table 6 that the proposed equation has a probability value (p > 0.05) meaning that there are no significant differences between the two equations.

**Table 6.** T test between two equations (Hussein-Otto).

Events	Differences		T	df	Sig. (2-tailed)
	Mean	Std. Deviation			
Discus	-0.11271	1.68306	-0.067	54	0.947
Javelin	-0.09500	2.66018	-0.036	30	0.972
Shotput	-0.37513	0.28373	-1.322	62	0.191

**Figure 2.**
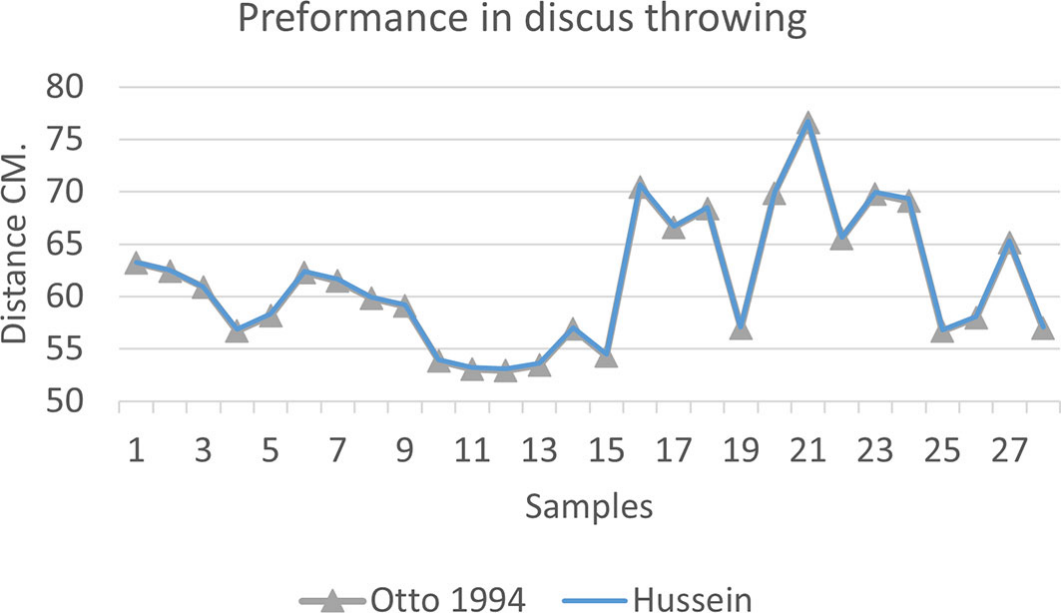
Horizontal distance of two equations in discus throwing.

**Figure 3.**
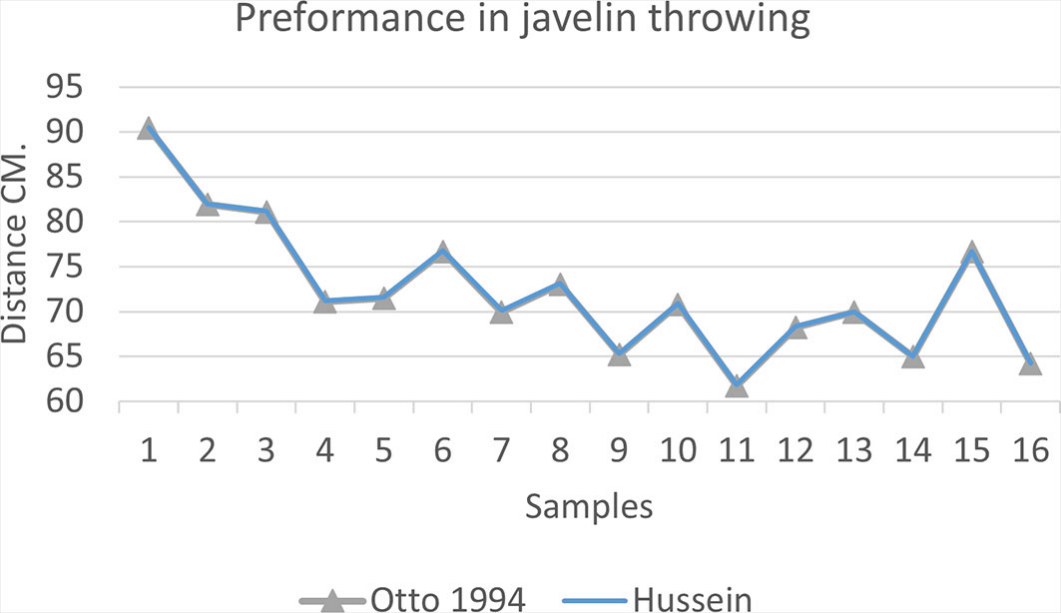
Horizontal distance of two equations in javelin throwing.

**Figure 4.**
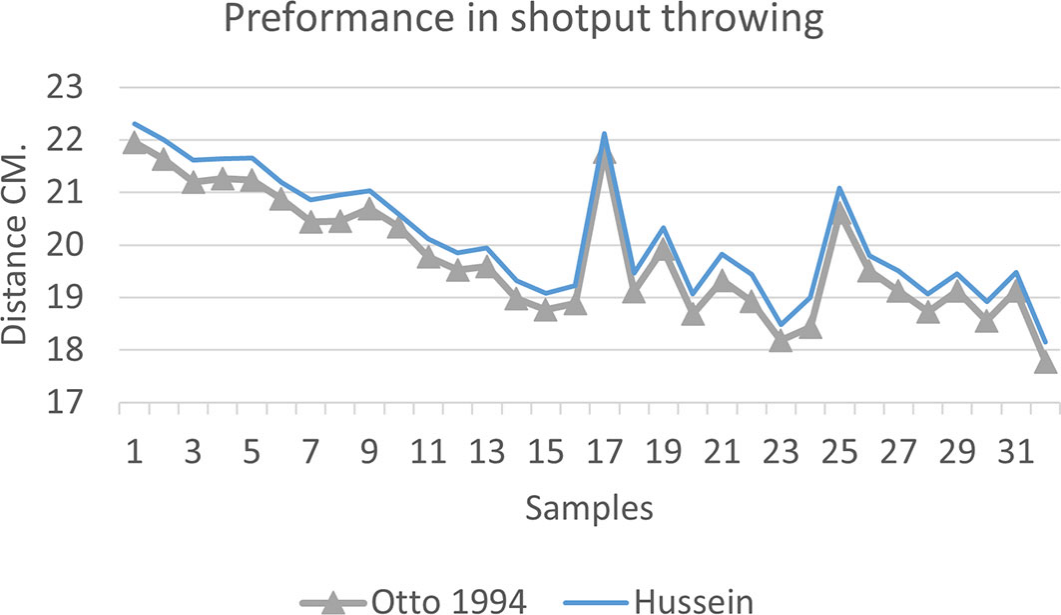
Horizontal distance of two equations in shotput throwing.

It is clear from Figures 2, 3 and 4 that the results were very close between the previous equation and the proposed equation, which indicates that our use of the proposed equation is possible, and it is a less complicated equation than the previous equation. As for Figure 4 we notice a difference between the two curves, reviewing Table 3, we find that the differences with the measurements made on the ground and the equation used were in favor of our study. The reason is due to the importance of velocity in the law, it is square, and since the weight of shotput is greater than the other tools, their velocity is less.

The figure shows the difference between the actual achievement that was measured on the ground in the discus throws event (Atlanta, 2009 Berlin 2006 Athena, 1966 male, and female), according to common law without calculating the height factor and by calculating the height factor in the two equations (Otto and Hussein).

The figure shows the difference between the actual achievement that was measured on the ground in the javelin throw event (Diego 2011 males and females), according to common law without calculating the height factor and by calculating the height factor in the two equations (Otto and Hussein).

Figure 7 shows the difference between the actual achievement that was measured on the ground in the Shotput throw event (2009 Berlin, 2011 Diego males and females), according to common law without calculating the height factor and by calculating the height factor in the two equations (Otto and Hussein).

**Figure 5.**
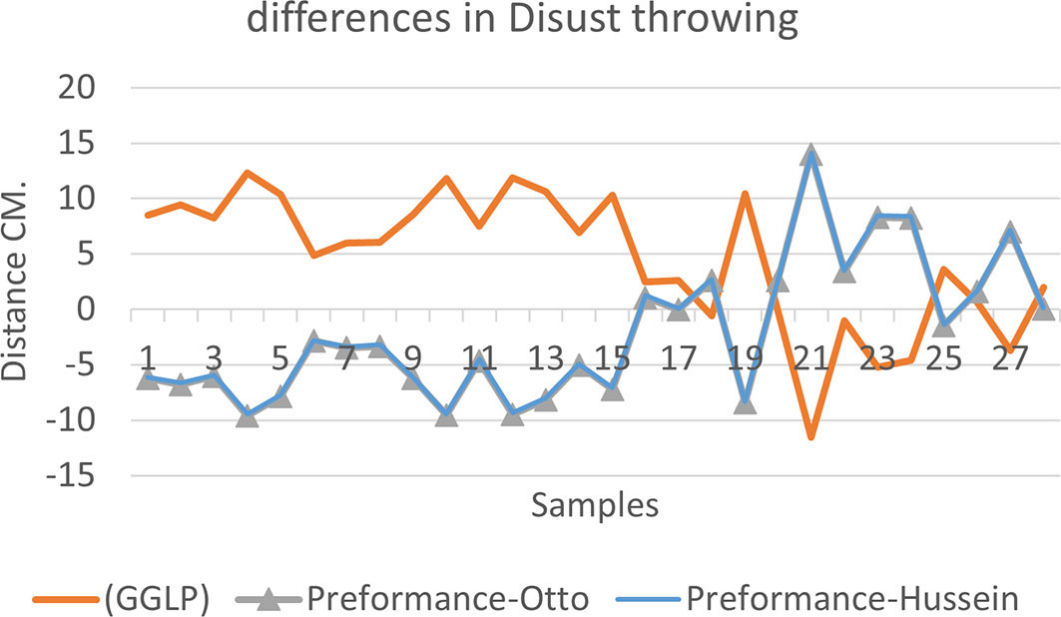
Difference between the actual achievement that was measured on the ground in the discus throwing.

**Figure 6.**
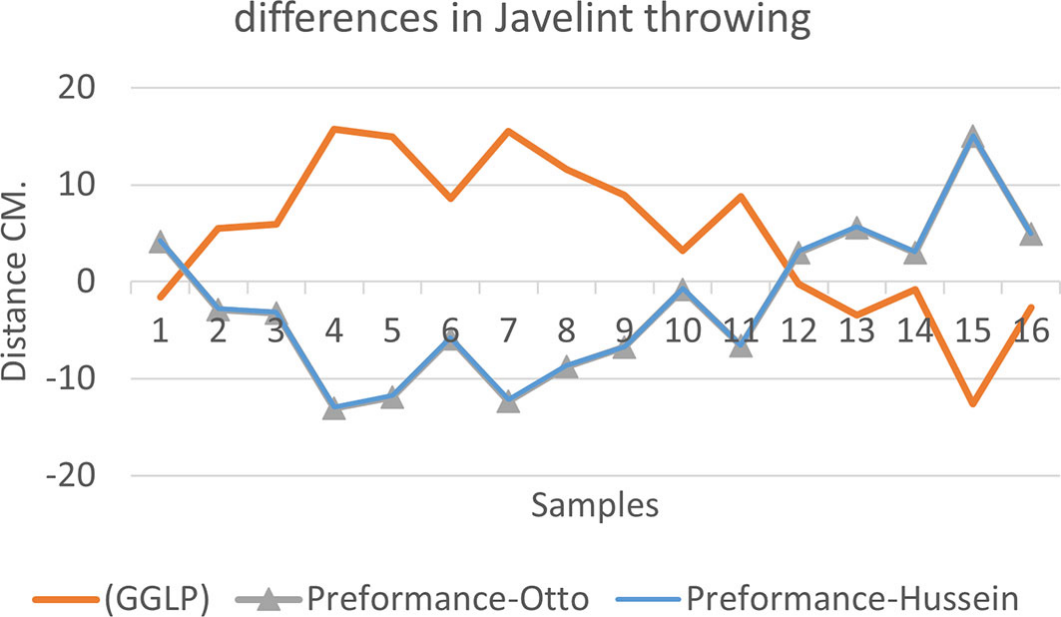
Difference between the actual achievement that was measured on the ground in the javelin throwing.

**Figure 7.**
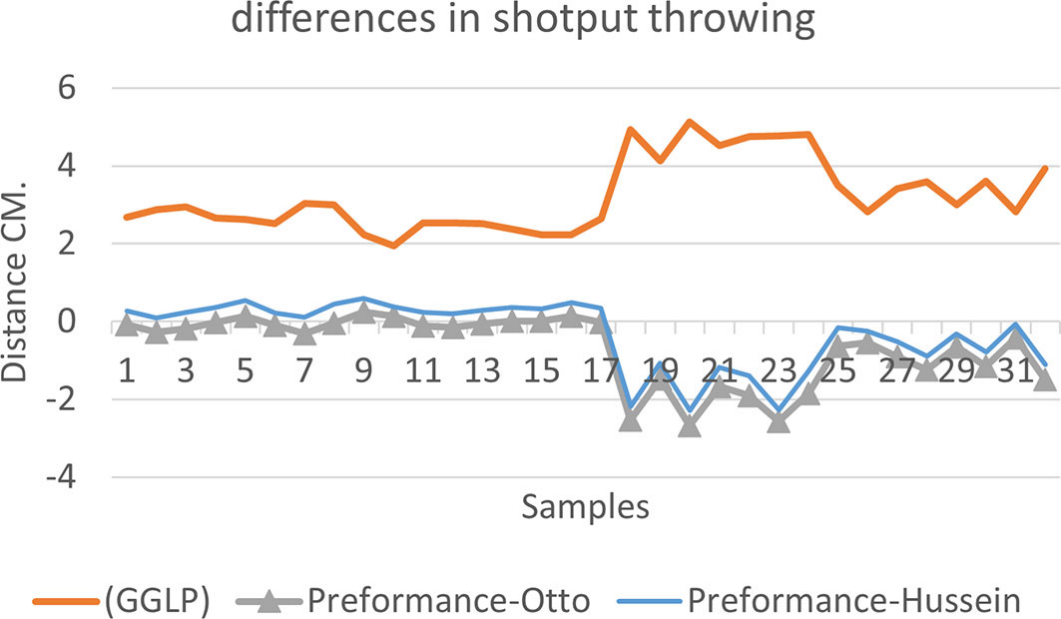
Difference between the actual achievement that was measured on the ground in the Shotput throwing.

We notice from Figures 5, 6 and 7 that when applying GGLP stripped of proposed equations, the samples will give us drawings that are inverted with the graphic curve for the same samples when we apply the two equations, which means that the proposed equation has the same components as the previous equation.

## Discussion

When analyzing the common law of projectiles, we see that the most critical factor on which the law depends is when the two launch points are equal. The launch (release) velocity, as it is squared, and although some tools are heavier than others, the law depends on the velocity, based on the power of the launch; the second factor is the angle of launch, and many experiments found that the release angle (45 degrees) is best when the launch and fall points are equal because this angle changes the magnitudes of the compounds the horizontal and vertical launch velocity is of secondary importance ( Hamill *et al*., 2015), as the global rate may be vertical more than this process leads to the tool gaining a vertical distance more than the horizontal, as occurs in the effectiveness of high jump.

Theoretically, achieving the best horizontal distance does not correspond to human bodies because they are designed for tools. Therefore, GGLP yields better results in throwing equipment compared to the long jump, or triple jump, and in all of these events, we notice that the center of gravity of the body emanates from a location different from the site of its descent, the shape or weight of the tool is not very large, and it also includes friction with the air or changing the direction of the wind. The law of events prevents recording some achievements due to the wind velocity that is expected to be the cause of the digital achievement. The critical factor is the height of the tool, and due to the lengths of the players being close, calculating the remaining distance is significant in this case, and as we observed in one of the figures, there must be an equation to calculate the remaining distance, because the common law will calculate the distance from the starting point to the point of fall parallel to the launch point.

The pervious equations put the same ( *v*) velocity of release twice in the equation, it is incorrect to put the same value and perform other calculations on it once, we followed the same procedure for the same ( *θ*) angle of release, we found that the sine and cosine is processed once mathematically.

The criticisms that can be directed at previous studies are that they considered the point at which the projectile reaches its initial level to be the end of the projectile, so they began with a new equation that was integrated with Galileo’s law. However, our study considered that the projectile’s reaching its launch level is the beginning of an angle that already exists, which is the launch angle. Considering that the height difference already exists, the proposed equation was formed according to this assumption.

In this study, we didn’t consider the case where the release point is lower than the landing point, as it occurs in High Jump or Basketball shooting, we need the horizontal displacement of performance events to calculate the horizontal displacement, this helps us control the three variables of the projectile for training purposes and to increase performance. However, we believe that the equation is still applicable in this case, because the terms used in the equation are the same. The equation, like its predecessors, did not address the weights of the tools used.

## Conclusions

Three important factors of projectiles were studied in three major throwing events of track and field events. These factors are (speed, angle, and height of the projectile). These factors were subjected to two types of equations for the purpose of finding the achievement, one of the equations is proposed in this study. The proposed equation was built on the basis that the angle of landing is the same as the angle of takeoff in the parabola, and from this we started to design the equation. This method prevented us from repeating the use of the three factors in the equation and the procedures of the computational operations again.

The results of the two equations were compared with the legal achievement that is measured on the ground as in competitions. We also used the Galileo Galilei Law of Projectiles as a second criterion to know the degree of convergence between the results of the two equations, as the Galileo Galilei Law of Projectiles is an important part of the equation designed by us.

It is important to note that the proposed equation and previous equations do not take into account the mechanical distance, i.e. the distance the thrower’s hand extends beyond the throwing boundary. This is because the achievements that are measured on the ground are from the outer edge of the throwing circle or the finish line.

The proposed equation has proven its credibility, statistically and graphically, and had high credibility when applied to (3) different activities in their velocity, weight and shape of throwing activities for track and field games, and the results are often closer to the real achievements.

The proposed equation is suitable for use as an alternative to Galileo’s law of projectiles with equal release and fall levels.

The importance of designing the equation comes from the fact that the three factors of the projectile can be important topics in the science of sports training for the development of achievement.

It is possible to conduct studies to compare with other equations, as we conducted a comparison with one equation. Such comparisons can also be conducted on events where the takeoff point is lower than the landing point (site of achievement calculation), as in high jump or shooting in basketball.

## Data Availability

Zenodo. A proposed equation for calculating the total horizontal distance in projectiles of varying launch and landing levels. DOI: https://doi.org/10.5281/zenodo.8309822. This project contains the following underlying data: Data.xlsx(data obtained from real sports competition sources for the three variables: velocity, angle, height, and horizontal measurement on the ground for events) ( Jung *et al*., 2012; Woen-Sik Chae, 2011; Panoutsakopoulos, 2008; Finals, 2011; Jung *et al*., 2012). Data are available under the terms of the Creative Commons Attribution 4.0 International license (CC-BY 4.0).
