# Supplementary material for: A proposed equation for calculating the total horizontal distance in projectiles of varying launch and landing levels
Source: F1000Res. 2024 Mar 13;12:1491. Originally published 2023 Nov 21. [Version 2] doi: 10.12688/f1000research.140934.2 (PMC10905019; doi:10.12688/f1000research.140934.2)
Supplement: Peer review history

# Reviewer report: Reviewer response for version 2

**DOI:** 10.5256/f1000research.163655.r331816

**Published:** 2024-10-16

**Authors:** Uday CH Hasan

**Affiliations:** 1 Department of Physical Education and Sports Sciences,, Al-Kitab University, Kirkuk, Iraq

**I approve this article**

Is the work clearly and accurately presented and does it cite the current literature?

Yes

If applicable, is the statistical analysis and its interpretation appropriate?

Yes

Are all the source data underlying the results available to ensure full reproducibility?

Yes

Is the study design appropriate and is the work technically sound?

Yes

Are the conclusions drawn adequately supported by the results?

Yes

Are sufficient details of methods and analysis provided to allow replication by others?

Yes

Reviewer Expertise:

Movement Analysis, Sport Biomechanics, Sports Science

I confirm that I have read this submission and believe that I have an appropriate level of expertise to confirm that it is of an acceptable scientific standard.

# Reviewer report: Reviewer response for version 2

**DOI:** 10.5256/f1000research.163655.r255536

**Published:** 2024-06-21

**Authors:** Yasir Najah

**Affiliations:** 1 Collage of physical education and sport sciences, University of Baghdad, Baghdad, Baghdad Governorate, Iraq

I have reviewed the researcher's revisions to the submitted research paper and found that they have completed the revisions and I approve this article.

Is the work clearly and accurately presented and does it cite the current literature?

Yes

If applicable, is the statistical analysis and its interpretation appropriate?

Yes

Are all the source data underlying the results available to ensure full reproducibility?

Partly

Is the study design appropriate and is the work technically sound?

Yes

Are the conclusions drawn adequately supported by the results?

Yes

Are sufficient details of methods and analysis provided to allow replication by others?

Yes

Reviewer Expertise:

Philosophy in physical education science Sports biomechanics and motor analysis

I confirm that I have read this submission and believe that I have an appropriate level of expertise to confirm that it is of an acceptable scientific standard.

# Reviewer report: Reviewer response for version 2

**DOI:** 10.5256/f1000research.163655.r255537

**Published:** 2024-04-22

**Authors:** Yarob Abdulbaqi Algaith

**Affiliations:** 1 Albasrah university, Basra, Iraq

I have reviewed the researcher's revisions to the submitted research paper and found that they have completed the revisions very well. The research is important for acceptance due to its significance in providing an equation for measuring horizontal distance.

Is the work clearly and accurately presented and does it cite the current literature?

Yes

If applicable, is the statistical analysis and its interpretation appropriate?

Yes

Are all the source data underlying the results available to ensure full reproducibility?

Yes

Is the study design appropriate and is the work technically sound?

Yes

Are the conclusions drawn adequately supported by the results?

Yes

Are sufficient details of methods and analysis provided to allow replication by others?

Yes

Reviewer Expertise:

A specialist in the field of sports biomechanics and kinematic analysis of sports games

I confirm that I have read this submission and believe that I have an appropriate level of expertise to confirm that it is of an acceptable scientific standard.

# Reviewer report: Reviewer response for version 2

**DOI:** 10.5256/f1000research.163655.r255538

**Published:** 2024-03-20

**Authors:** Basman Abdul Jabbar

**Affiliations:** 1 Physical Education and Health Sciences (ISPEHS), Edinburgh University Institute for Sport,, Edinburgh, Scotland, UK

I approve this article

Is the work clearly and accurately presented and does it cite the current literature?

Yes

If applicable, is the statistical analysis and its interpretation appropriate?

I cannot comment. A qualified statistician is required.

Are all the source data underlying the results available to ensure full reproducibility?

Yes

Is the study design appropriate and is the work technically sound?

Yes

Are the conclusions drawn adequately supported by the results?

Yes

Are sufficient details of methods and analysis provided to allow replication by others?

Yes

Reviewer Expertise:

Sports Biomechanics and Motion Analysis

I confirm that I have read this submission and believe that I have an appropriate level of expertise to confirm that it is of an acceptable scientific standard.

# Reviewer report: Reviewer response for version 1

**DOI:** 10.5256/f1000research.154342.r224631

**Published:** 2024-06-29

**Authors:** Uday CH Hasan

**Affiliations:** 1 Department of Physical Education and Sports Sciences,, Al-Kitab University, Kirkuk, Iraq

Dear Helena

 The Editorial Team, F1000Research

 I hope you are fine.

 I would like to thank the editorial board for choosing us to be reviewer in "F1000Research"

 I accept the submission with the possibility of considering the proposed equation as a patent due to its ease of application and accuracy of calculation, and to be adopted in biomechanics curricula, I suggest that the following should be clarified:

 1- Is the equation valid for calculating the horizontal distance at different levels (difference in the initial height and final height) in general, or is it valid only if the initial height is higher than the final height?

 2- It is necessary to take into account the effectiveness of the javelin throw to calculate the initial and final height, especially the final height that is not at the level of the ground. Therefore, it is necessary to clarify the following in terms of the effectiveness of the javelin throw: total distance, achievement distance, and mechanical distance.

 To sum up, I endorse the article.

Is the work clearly and accurately presented and does it cite the current literature?

Yes

If applicable, is the statistical analysis and its interpretation appropriate?

Yes

Are all the source data underlying the results available to ensure full reproducibility?

Yes

Is the study design appropriate and is the work technically sound?

Yes

Are the conclusions drawn adequately supported by the results?

Yes

Are sufficient details of methods and analysis provided to allow replication by others?

Yes

Reviewer Expertise:

Movement Analysis, Sport Biomechanics, Sports Science

I confirm that I have read this submission and believe that I have an appropriate level of expertise to confirm that it is of an acceptable scientific standard.

# Reviewer report: Reviewer response for version 1

**DOI:** 10.5256/f1000research.154342.r247956

**Published:** 2024-02-29

**Authors:** Basman Abdul Jabbar

**Affiliations:** 1 Physical Education and Health Sciences (ISPEHS), Edinburgh University Institute for Sport,, Edinburgh, Scotland, UK

Researcher used  the derivations through the projectiles equation to find the horizontal distance.

 The main equation that the researcher processed was Galileo’s equation for projectiles, and the research sample was appropriate by choosing three different throwing events in the arena and field. This is an innovative way to address the measurement problem in this type of activity.The main equation that the researcher processed was Galileo’s equation for projectiles, and the research sample was appropriate by choosing three different throwing events in the arena and field.

 The method was innovative to address the measurement problem in this type of activity. I suggested the possibility of comparison with more than statistical treatment of the same variables, or they could be added to the recommendations for the purpose of benefiting for the future.

Is the work clearly and accurately presented and does it cite the current literature?

Yes

If applicable, is the statistical analysis and its interpretation appropriate?

I cannot comment. A qualified statistician is required.

Are all the source data underlying the results available to ensure full reproducibility?

Yes

Is the study design appropriate and is the work technically sound?

Yes

Are the conclusions drawn adequately supported by the results?

Yes

Are sufficient details of methods and analysis provided to allow replication by others?

Yes

Reviewer Expertise:

Sports Biomechanics and Motion Analysis

I confirm that I have read this submission and believe that I have an appropriate level of expertise to confirm that it is of an acceptable scientific standard.

# Response

**Published:** 2024-02-29

**Authors:** hussein omer

**Affiliations:** Physical Education, University of Al-Qadisiyah, Diwaniyah, Al-Qādisiyyah Governorate, Iraq

The reviewer's proposal is clear, and a comment will be made on the possibility of using advanced statistical treatments for the purpose of making better use of the results of this study.

# Reviewer report: Reviewer response for version 1

**DOI:** 10.5256/f1000research.154342.r224629

**Published:** 2024-02-14

**Authors:** Yarob Abdulbaqi Algaith

**Affiliations:** 1 Albasrah university, Basra, Iraq

The study is very well done and provides a suitable and easy way to measure the horizontal distance of a projectile in non-symmetrical levels. I have conducted an experiment on it and it has been shown to be effective and achieve results that are closer to the real distance and in a better way than what is available in the results of the computational averages in the tables. However, there are some values in the tables for the minimum and maximum columns that need to be reviewed and there are errors in them, especially in the differences between the results of the equations. I also recommend that an equation be found to calculate the time that accompanies such an equation. This is generally necessary, as the time must be calculated in an easier way than what is available to us currently. However, the required of the study has been achieved, especially in such sporting events, which is to achieve the best distance only regardless of the time.

Is the work clearly and accurately presented and does it cite the current literature?

Yes

If applicable, is the statistical analysis and its interpretation appropriate?

Yes

Are all the source data underlying the results available to ensure full reproducibility?

Yes

Is the study design appropriate and is the work technically sound?

Yes

Are the conclusions drawn adequately supported by the results?

Yes

Are sufficient details of methods and analysis provided to allow replication by others?

Yes

Reviewer Expertise:

A specialist in the field of sports biomechanics and kinematic analysis of sports games

I confirm that I have read this submission and believe that I have an appropriate level of expertise to confirm that it is of an acceptable scientific standard, however I have significant reservations, as outlined above.

# Response

**Published:** 2024-04-14

**Authors:** hussein omer

**Affiliations:** Physical Education, University of Al-Qadisiyah, Diwaniyah, Al-Qādisiyyah Governorate, Iraq

In V2, the comments of all peers were amended or included...with appreciation

# Reviewer report: Reviewer response for version 1

**DOI:** 10.5256/f1000research.154342.r224628

**Published:** 2024-02-14

**Authors:** Yasir Najah

**Affiliations:** 1 Collage of physical education and sport sciences, University of Baghdad, Baghdad, Baghdad Governorate, Iraq

Dear Editors of F1000Research,

 Regarding the evaluation of the attached article, there are some positive and negative points that have been found and I will tackle each point according to its priority:

1. The idea of proposing an equation for calculating varied levels of launching and landing is smart and creative specially because the researcher used a well – known geometrical system for simplifying this types of projectile’s equation a matter that makes this equation more simple to use and less complicated.
2. The proposed equation cannot be generalized just because it was applied on three events in athletics for the following reasons:

1. All three events selected for the experiment have a higher launching level compared to the landing level and hence a question arises; should the same procedures be followed when the launching level is lower than landing level since the base of the drawn triangle will be in the launching side not the landing side? I would have preferred if the experiment covered two types of projectiles with different levels.
2. According to my experience in biomechanics and gymnastics, within the same level that the researcher studied we find it difficult for most gymnasts to land from the horizontal bar with a smaller angle than landing angle and this does not match the researcher’s hypothesis concerning the proposed equation. The same is true concerning very high projectiles because their launching angle is smaller than their landing angle. In the first figure we find that angle (B) is smaller than angle (F) and this is the reason for differences in results otherwise the result should be identical according to the geometrical system. Therefor I would have preferred conducting more experiments to cover all variances so as to reach the final goal of generalizing this equation.

   

1. In the events that the researcher studied there are two distances; the actual throwing distance that is calculated manually according to the equation of the sport and the second distance is the mechanical distance calculated using projectile equation thus there are always differences in these distances based on launching point, calculation start line, landing point, and the last mark left on the ground. In this point it is difficult to decide which one is more accurate. Thus I propose, in order to generalize this equation, making further studies starting from the official distance for projectiles that are the same as the concluded mechanical distance calculated using projectile equation.
2. The researcher recommended generalizing the proposed equation on throwing events in athletics only hoping for further generalization after conducting more researches on these points.

Thank you for your trust

 Professor Yasser Nejah (PhD)    

Is the work clearly and accurately presented and does it cite the current literature?

Yes

If applicable, is the statistical analysis and its interpretation appropriate?

Yes

Are all the source data underlying the results available to ensure full reproducibility?

Partly

Is the study design appropriate and is the work technically sound?

Yes

Are the conclusions drawn adequately supported by the results?

Yes

Are sufficient details of methods and analysis provided to allow replication by others?

Yes

Reviewer Expertise:

Philosophy in physical education science Sports biomechanics and motor analysis

I confirm that I have read this submission and believe that I have an appropriate level of expertise to confirm that it is of an acceptable scientific standard.

## References

[1] : applied biomechanics concepts and conections. *thomson wordsworth* .2008;

# Response

**Published:** 2024-02-14

**Authors:** hussein omer

**Affiliations:** Physical Education, University of Al-Qadisiyah, Diwaniyah, Al-Qādisiyyah Governorate, Iraq

The reviewer’s proposal is clear, and a simple comment will be made while explaining the equation, stating that it is designed for these three activities and may be suitable for application to other activities, and may be suitable for application at launch and landing levels opposite to what is in the equation.
